# 

**Published:** 1908-03

**Authors:** 


PUBLISHED MONTHLY. ATLANTA SUBSCRIPTION $1.00

### JOURNAL-RECORD OF MEDICINE

LIBRARY
MAR. 16 1908

Successor to { Atlanta Medical and Surgical Journal, Established 1855.
and Southern Medical Record, Established 1870. } 52d Year

Vol. X. ATLANTA, GA., MARCH, 1908. No. 12

